# Acute Unilateral Vision Loss in a 61-Year-Old Woman: Distinguishing Non-arteritic Anterior Ischemic Optic Neuropathy (NAION) From Optic Neuritis and Papilledema

**DOI:** 10.7759/cureus.110411

**Published:** 2026-06-07

**Authors:** Elizaveta Yagudaeva, Javokhir Juraev, Diyora Khakimjonova, Mokhiryam Alieva

**Affiliations:** 1 School of Medicine, Central Asian University, Tashkent, UZB; 2 Department of Ophthalmology, AKFA Medline University Hospital, Tashkent, UZB

**Keywords:** c-reactive protein, giant cell arteritis, hypertension, naion, non-arteritic anterior ischemic optic neuropathy, optical coherence tomography, optic disc edema

## Abstract

Within the population over age 50, non-arteritic anterior ischemic optic neuropathy (NAION) represents a primary etiology for acute, unilateral visual impairment. It shares common features with conditions like optic neuritis and papilledema, making accurate diagnosis difficult. A 61-year-old woman came to AKFA Medline University Hospital with sudden, painless vision loss in her left eye that began four days prior. Her medical history included high blood pressure. An eye examination revealed swelling of the optic disc with blurred edges and decreased vision in her left eye. Evaluation via optical coherence tomography (OCT) revealed prominent edema within the retinal nerve fiber layer, indicating involvement of the acute optic nerve head. A magnetic resonance imaging scan of the brain and orbits ruled out intracranial problems and demyelinating diseases. Laboratory results indicated that the erythrocyte sedimentation rate (ESR) did not deviate from normal limits; however, a marginal rise in C-reactive protein was noted, with no clinical evidence to support a diagnosis of systemic vasculitis or giant cell arteritis. Considering her clinical presentation, imaging results, and the exclusion of other potential causes, a diagnosis of non-arteritic anterior ischemic optic neuropathy was established. She received conservative treatment focused on controlling vascular risk factors, and follow-up visits showed stable vision with some improvement in optic disc swelling. This case emphasizes the need for a thorough diagnostic method that combines clinical evaluation, imaging, and lab tests. This approach is crucial for distinguishing NAION from other causes of acute optic disc edema, helping to prevent misdiagnosis and improper treatment.

## Introduction

Among the geriatric population, acute visual impairment is frequently attributed to anterior ischemic optic neuropathy (AION), particularly in individuals exceeding 50 years of age [1,2]. It has two main types: arteritic and non-arteritic. Non-arteritic AION (NAION) occurs more often and is linked to risk factors like high blood pressure. Telling NAION apart from other causes of optic disc swelling, such as optic neuritis and papilledema, is a clinical challenge because of similar signs [3,4]. A correct diagnosis is crucial to guide treatment and prevent unnecessary interventions. We report a case of suspected NAION in a patient with high blood pressure. This case highlights the diagnostic steps and key features that help differentiate it from other optic neuropathies.

## Case presentation

A 61-year-old woman experienced sudden, painless vision loss in her left eye that started four days before her visit. She reported no pain with eye movement, headaches, jaw pain, scalp tenderness, or other systemic symptoms. Her medical history included high blood pressure. She had no history of diabetes, autoimmune diseases, or recent infections.

Ocular assessment revealed a best-corrected visual acuity (BCVA) of 20/20 in the right eye, whereas the left eye demonstrated a significant reduction to 20/100. Following refractive correction, the left eye improved to 20/40. Pupillary assessment revealed a sluggish light reflex in the left eye with a positive relative afferent pupillary defect (RAPD). Color vision testing via Ishihara plates demonstrated an acute deficit in the left eye (3/11 plates), while the right eye was completely intact (11/11 plates). Intraocular pressure was normal in both eyes. The slit-lamp exam of the right eye showed no issues. In the left eye, there was mild redness of the conjunctiva, but the anterior segment structures were otherwise normal. The fundoscopic examination revealed pale, diffuse optic disc edema with blurred margins restricted strictly to the left eye, along with some intraretinal bleeding (Figure 1). The right optic disc appeared normal. An automated Humphrey Visual Field (HVF) 30-2 protocol was attempted; however, the results were highly unreliable due to the patient's poor central fixation and advanced visual acuity loss in the left eye.

**Figure 1:**
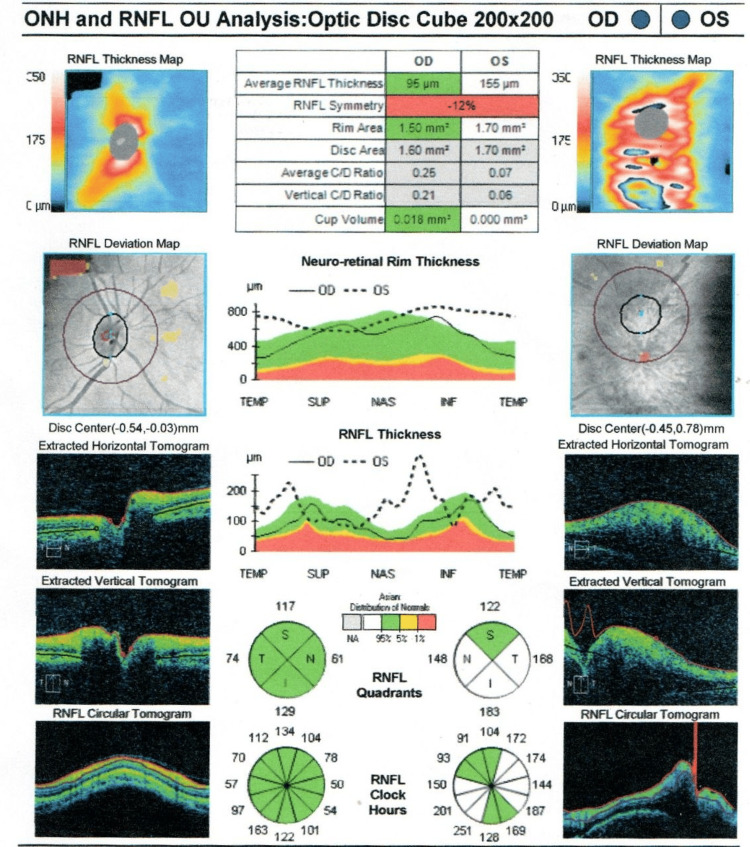
Imaging of the optic nerve head (ONH) and retinal nerve fiber layer (RNFL) via spectral-domain OCT. The optic disc cube 200 x 200 acquisition protocol was utilized for bilateral assessment. OCT: optical coherence tomography; OU: oculus uterque (both eyes); OD: oculus dexter (right eye); OS: oculus sinister (left eye); C/D: cup-to-disc ratio; TEMP: temporal; SUP: superior; NAS: nasal; INF: inferior; S: superior; T: temporal; I: inferior; N: nasal.

Optical coherence tomography (OCT) showed swelling in the retinal nerve fiber layer of the affected eye (Figures 2, 3). An MRI of the brain and orbits showed no signs of intracranial problems or optic nerve damage (Figure 4). Lab tests, including a complete blood count, biochemical analysis, and coagulation profile, were all normal. The erythrocyte sedimentation rate was normal, while C-reactive protein (CRP) was slightly elevated, with no signs of systemic vasculitis. The mildly elevated CRP initially raised suspicion for an inflammatory or arteritic process. However, the absence of systemic markers for giant cell arteritis such as jaw claudication, scalp tenderness, or a high ESR allowed for a more focused investigation into non-arteritic causes. Metabolic serum panels revealed mild hypercholesterolemia (total cholesterol: 224 mg/dL, low-density lipoprotein (LDL): 142 mg/dL), while triglycerides and high-density lipoprotein (HDL) were within normal physiological ranges. The glycated hemoglobin (HbA1c) level was 6.2%, indicating impaired glucose metabolism. Therefore, we referred the patient to an endocrinologist for further consultations.

**Figure 2:**
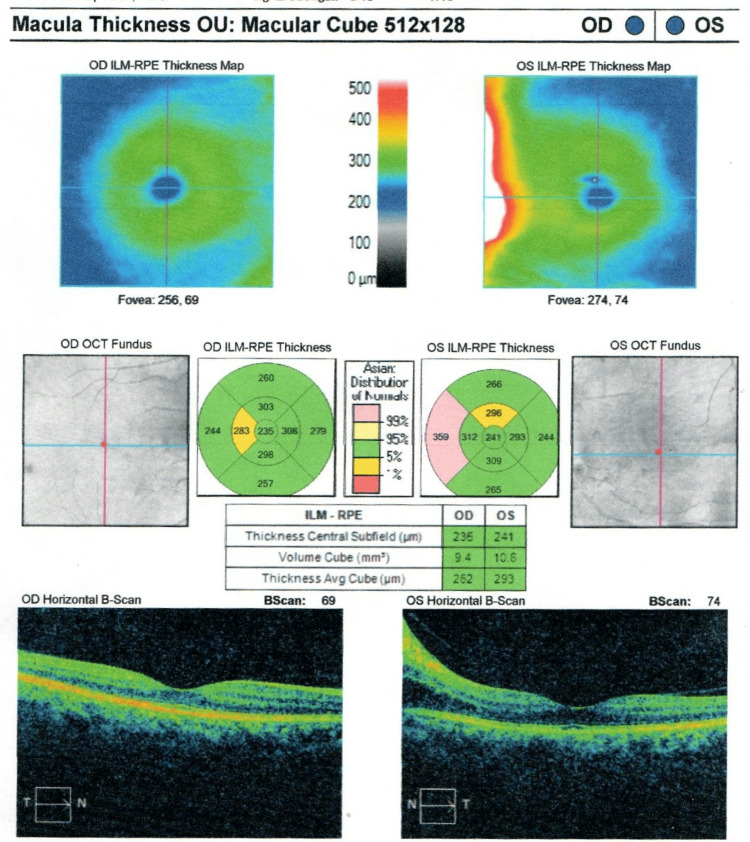
Optical coherent tomography (OCT) of the macula. Spectral-domain OCT (macular cube 512 x 128) was performed in both eyes. OU: oculus uterque (both eyes); OD: oculus dexter (right eye); OS: oculus sinister (left eye); ILM-RPE: internal limiting membrane-retinal pigment epithelium.

**Figure 3:**
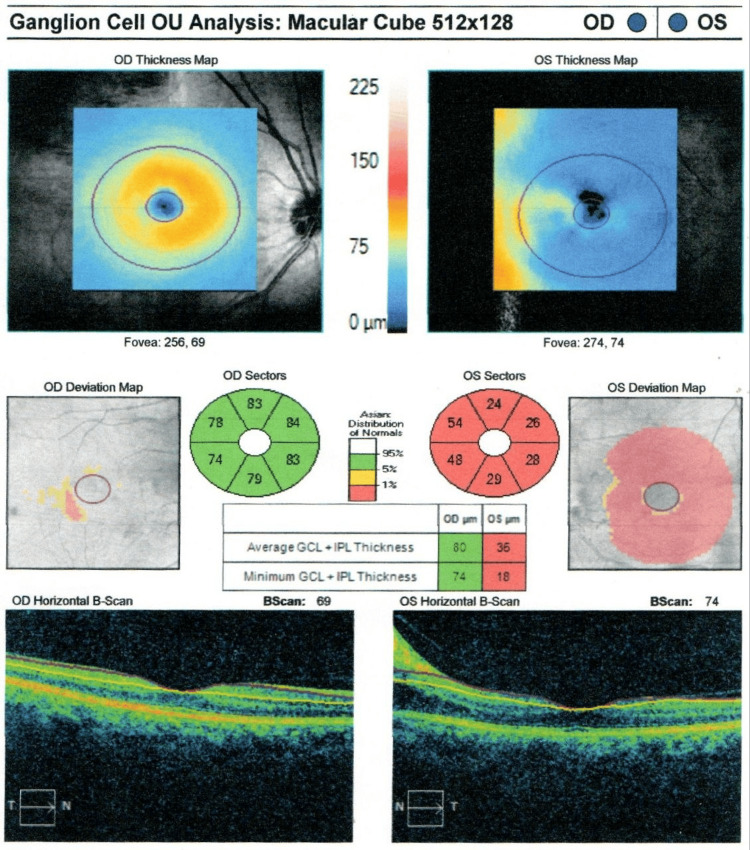
Spectral-domain optical coherence tomography (OCT) ganglion cell analysis (macular cube 512 × 128) performed in both eyes, demonstrating optic disc edema with retinal nerve fiber layer changes in the left eye, with partial extension toward the macular region. OU: oculus uterque (both eyes); OD: oculus dexter (right eye); OS: oculus sinister (left eye); GCL: ganglion cell layer; IPL: inner plexiform layer.

**Figure 4:**
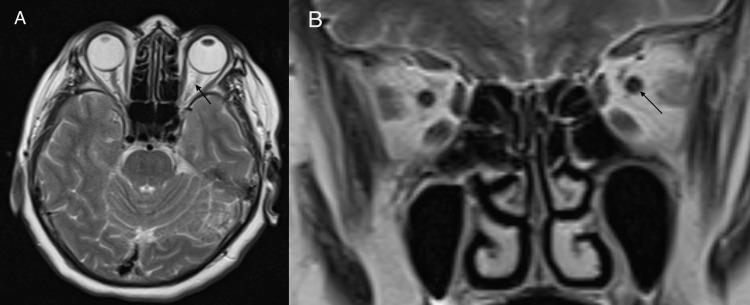
Magnetic resonance imaging (MRI) of the orbits. (A) Axial T2-weighted turbo spin echo (TSE) sequence and (B) coronal T2-weighted sequence. The arrows indicate the left optic nerve, which demonstrates normal caliber and isointense signal. These findings confirm the absence of orbital masses, extraocular muscle enlargement, or inflammatory enhancement.

Considering the painless vision loss, optic disc swelling, vascular risk factors, and ruling out other causes, non-arteritic anterior ischemic optic neuropathy was concluded to be the most likely diagnosis. Following the diagnosis, the patient was managed conservatively with optimization of vascular risk factors. Her antihypertensive regimen was adjusted in consultation with internal medicine, while lifestyle modification, dietary counseling, and lipid-lowering therapy were initiated to address impaired glucose metabolism (HbA1c 6.2%) and hypercholesterolemia. The patient was followed for six months with serial neuro-ophthalmologic evaluations. At one month, fundoscopic examination demonstrated partial resolution of optic disc edema, accompanied by a slight improvement in color vision from 3/11 to 5/11 Ishihara plates. By three months, optic disc edema had resolved with mild temporal disc pallor, and repeat OCT showed regression of retinal nerve fiber layer swelling. A repeat Humphrey Visual Field examination revealed a stable inferior altitudinal defect in the affected eye. At the final six-month follow-up, left-eye BCVA had stabilized at 20/70 (20/30 with pinhole correction), color vision improved to 6/11 Ishihara plates, intraocular pressures remained normal bilaterally, and no new visual or systemic symptoms were reported. The right eye remained unaffected throughout the follow-up period.

## Discussion

In the differential diagnosis of sudden sight loss for patients over 50, NAION is frequently identified as the leading cause. It usually appears as a quick, painless loss of vision in one eye, accompanied by swelling of the optic disc [5]. This condition occurs as a result of reduced blood flow to the optic nerve head, which is nurtured by the posterior ciliary circulation [6]. It is closely linked to vascular risk factors like high blood pressure, diabetes, and low blood pressure at night.

In this patient, we detected a sudden, painless loss of vision in one eye, swelling of the optic disc, and a history of high blood pressure, all pointing to NAION. Optical coherence tomography confirmed swelling in the retinal nerve fiber layer, indicating ischemia in the optic nerve head [7]. Magnetic resonance imaging ruled out compressive and demyelinating issues.

One of the primary diagnostic hurdles was the exclusion of arteritic anterior ischemic optic neuropathy, specifically giant cell arteritis (GCA). Although laboratory findings showed a marginal increase in C-reactive protein (CRP), the erythrocyte sedimentation rate (ESR) remained within the normal range. CRP can be more sensitive than ESR in detecting GCA; however, without systemic symptoms like jaw pain, scalp tenderness, fever, or weight loss, and considering the patient’s vascular risk factors, arteritic AION seemed unlikely. This shows that mild CRP elevation can appear in non-arteritic ischemic optic neuropathy and should be carefully assessed in context.

The differential diagnosis also included optic neuritis and papilledema. Possibility of an optic neuritis was excluded with the patient's age, lack of eye pain during movement, and absence of demyelinating lesions on MRI [8,9]. Papilledema was discounted because of the unilateral nature, the lack of increased intracranial pressure on imaging, and the absence of symptoms like headaches or brief visual disturbances.

When evaluating acute unilateral optic disc edema, it is vital to screen for secondary systemic triggers and rule out malignant hypertensive crises. Obstructive sleep apnea (OSA) is a well-documented risk factor for NAION, as nocturnal hypoxia and systemic hypotension can compromise optic nerve head perfusion; similarly, certain medications like phosphodiesterase-5 inhibitors have been linked to ischemic events. In our patient, a thorough history confirmed the absence of these high-risk exposures. Furthermore, malignant hypertension was safely excluded; the patient's blood pressure was stable at 145/88 mmHg without an acute hypertensive crisis (>180/120 mmHg), and her fundoscopic findings were strictly unilateral, whereas malignant hypertension characteristically presents with bilateral grade IV hypertensive retinopathy and disc swelling.

Overall, combining clinical signs, neuroimaging, lab results, and OCT findings led to a diagnosis of NAION. This case highlights the need for a thorough diagnostic approach in patients with sudden optic disc swelling to distinguish ischemic optic neuropathy from inflammatory, demyelinating, and intracranial issues. A systematic evaluation should include (1) assessment of pain, (2) fundoscopic examination, (3) MRI of the brain and orbits, and (4) inflammatory markers. This is crucial to avoid misdiagnosis and inappropriate treatment. For acute unilateral optic disc swelling, a structured approach is advisable: painful vision loss with demyelinating lesions suggests optic neuritis; bilateral disc swelling with increased intracranial pressure points to papilledema; and sudden, painless vision loss with vascular risks and normal imaging aligns most closely with NAION. The role of systemic corticosteroids in NAION remains highly controversial. While mandatory for arteritic AION (giant cell arteritis) to prevent devastating bilateral vision loss, major clinical data show no statistically significant long-term visual benefits for non-arteritic etiologies. Given our patient’s non-arteritic profile (normal ESR and absent constitutional symptoms), systemic steroid therapy was intentionally withheld. This decision was further supported by her metabolic risk profile; exposing a patient with pre-existing hypertension and newly identified impaired glucose tolerance (HbA1c, 6.2%) to high-dose corticosteroids carries a high risk of acute metabolic destabilization.

## Conclusions

Finding the right cause of acute unilateral vision loss in an older patient is a challenge that requires a high degree of diagnostic caution. While non-arteritic anterior ischemic optic neuropathy (NAION) is a common culprit in patients over 50, its clinical similarity with optic neuritis and papilledema can lead to diagnostic confusion. In this case, transitioning from a broad differential to a definitive diagnosis of NAION was achieved by leaning on multimodal imaging. High-resolution optical coherence tomography (OCT) provided objective evidence of ischemia through retinal nerve fiber layer (RNFL) and ganglion cell complex analysis, while magnetic resonance imaging (MRI) was vital in ruling out aggressive inflammatory or demyelinating processes.

A key takeaway from this case is the "CRP trap." A mild elevation in inflammatory markers can be a distracting finding; however, when systemic symptoms of giant cell arteritis are absent and the erythrocyte sedimentation rate (ESR) remains normal, such markers likely reflect a non-specific response rather than an arteritic process. Ultimately, by accurately identifying the ischemic nature of the vision loss and addressing the patient’s underlying vascular risks, specifically hypertension and impaired glucose metabolism, we were able to provide a clear prognosis and a targeted management plan. This case serves as a reminder that in neuro-ophthalmology, the correct diagnosis is found at the intersection of advanced technology and careful clinical reasoning.
